# Body weight prediction of Belgian Blue crossbred using random forest

**DOI:** 10.5455/javar.2024.k763

**Published:** 2024-03-31

**Authors:** Lisa Praharani, Chalid Talib, Diana Andrianita Kusumaningrum, Yeni Widiawati, Santiananda Arta Asmarasari, Supardi Rusdiana, Zultinur Muttaqin, Ria Sari Gail Sianturi, Elizabeth Wina, Endang Sopian, Aqdi Faturahman Arrazy, Umi Adiati, Ferdy Saputra

**Affiliations:** 1 Research Center for Animal Husbandry, Research Organization for Agriculture and Food, National Research Innovation Agency of the Republic of Indonesia, Bogor, Indonesia; 2 Indonesian Research Institute for Animal Production, Bogor, Indonesia

**Keywords:** Random forest, Belgian blue crossbred, body weight, morphometrics

## Abstract

**Objective:**

The aim of this study was to predict the body weight (BW) of a Belgian Blue X Friesian Holstein (BB X FH) crossbred in Indonesia based on morphometrics using random forest.

**Materials and Methods:**

A total of 26 BB X FH crossbreds were observed for BW, chest weight (CW), body length (BL), hip height (HH), wither height (WH), and chest girth (CG) from 0, 30, 60, 90, 120, 150, 180, 210, 240, 270, and 300 days of age. Stepwise regression and random forest were performed using R 3.6.1.

**Results:**

The random forest results show that CG is an important variable in estimating BW, with an important variable value of 24.49%. Likewise, the results obtained by stepwise regression show that CG can be an indicator of selection for the BB X FH crossbred. The *R* squared value obtained from the regression is 0.83, while the *R* squared value obtained from the random forest (0.86) is greater than the regression.

**Conclusion:**

In conclusion, random forest produces a better model than stepwise regression. However, a good simple equation to use to estimate BW is CG.

## Introduction

Belgian Blue cattle is a double-muscled cattle originating from Belgium. This cattle has an 11-nucleotide base deletion in the myostatin gene. In Indonesia, these cattle were bred by crossbreeding with other *Bos taurus* or local Indonesian cattle [1,2]. The Belgian Blue cannot adapt well to warm regions; therefore, the BB is crossed with other local cattle, one of which is the Friesian Holstein (FH), resulting in better performance than other crossbreds [3]. Preliminary research on Belgian Blue X Friesian Holstein (BB X FH) crossbreds for body weight (BW), morphology, and survivability has been conducted in Indonesia [4,5]. However, no research has been conducted to predict the growth rate of BB X FH.

Machine learning (ML) is an algorithmic development that is widely used to solve various problems. ML is widely used in animal science for predicting many live BWs [6], such as in chickens [7] and cattle [8], predicting milk yield [9], and precision nutrition [10]. ML algorithms are classified into three types of learning: reinforcement learning, unsupervised learning, and supervised learning [11]. Random forests in animal science are used to classify breeds, predict BW, and identify important single nucleotide polymorphisms (SNPs) [12–14].

Random forest is a popular ML algorithm that is widely used in classification and regression and has various advantages and high accuracy [15]. Therefore, this study aimed to predict the BW of a BB X FH crossbred in Indonesia based on morphometrics using random forest.

## Materials and Methods

### Ethical approval

The experimental procedures were carried out following the guidelines established by the Ministry of Agriculture of Indonesia. The Indonesian Agency for Agricultural Research and Development approved the procedures (Balitbangtan/Balitnak/Rm/06/2021).

### Animals

A total of 26 Belgian Blue X FH crossbreds were observed for BW, chest weight (CW), body length (BL), hip height (HH), wither height (WH), and chest girth (CH) from 0, 30, 60, 90, 120, 150, 180, 210, 240, 270, and 300 days of age. BW was measured in kg using the animal scale for cattle. Morphometric variables were measured using animal tape (Rondo). The experiment was carried out at the Indonesian Research Institute for Animal Production in Bogor, Indonesia.

### Feeding and management

The animals were kept in individual pens equipped with feeders and automatic drinking water. The feeder and drinking water tanks were cleaned every day. The cage was built from iron pipes on each side with concrete material for the floor. The roof of the cage was made from asbestos. From the age of 1 to 21, the animals were fed milk for about 2 l per head per day, then continuously increased up to 5 l per head per day. Starting at the age of 90 days, the animals were offered a mix of fresh chopped Napier grass and concentrate up to 200 g per head per day, which were offered after the calves received milk. Then, at the weaning age (about 5 months of age), the animals were fed a mixture of freshly chopped Napier grass and concentrate for about 2 kg. The legume leaves (*Gliricidia sepium*) were also offered to each animal, up to 1 kg per head per day. After that, all the animals received daily feed, which consisted of mixed fresh chopped Napier grass and legume leaves (20–25 kg per head per day) and concentrates (3 kg per head per day).

### Analysis data

Descriptive statistics, stepwise regression, and random forest were performed using R 3.6.1 [16]. The goodness-of-fit of the regression models was assessed using the coefficient of determination (R2) and Akaike’s information criterion (AIC). Stepwise regression was used to develop equations to predict BW from morphometric traits and age. In this study, three machine-learning methods have been used for estimating the weight of crossbreds during the first 300 days of life using body measurements.

## Results and Discussion

Based on the results of descriptive statistics, the BW and morphometric size of males are greater than those of females. In most animals, males are larger than females and are affected by testicular secretions (testosterone and its metabolites) [17]. The BW and morphometric size are presented in Tables 1 and 2. BW at 300 days of BB X FH was greater than that of Boran beef cattle from Kenya at 365 days of age [18]. This shows that *B. taurus* has better BW performance than *Bos indicus*. Based on the results of stepwise regression, BL and CG are the best models for estimating the BW of crossbreds (Table 3). However, CG is a simple and good variable for estimating BW. On the other hand, rump height has a high direct effect value in predicting BW in Nguni cattle [19]. In Hereford cows, metacarpus girth and backside half-girth are important variables in estimating live weight [20]. Therefore, CG and BL can be selection criteria for crossbreeding between Belgian Blue and FH. Head length contributed to 88% of the variation in male BW in South African Kalahari Red goats [21].

Based on the results of random forest, the variable importance values in this study were 21.88%, 20.88%, 24.49%, 23.28%, and 20.49% for WH, BL, CG, HH, and CW, respectively. These results support the previous stepwise regression analysis, where CG was an important variable for BB X FH cattle. An increase in node purity (IncNodePurity) denotes a change in the homogeneity of the groups formed by the trees. Based on the random forest results, it is known that two variables have optimal results in predicting BW in crossbreds; this can be seen from the high *R*^2^ value, while the Root Mean Square Error (RMSE) and Mean Absolute Error (MAE) values are the smallest (Table 4). In this study, we found random forests produced better prediction performance. However, the animal samples are very limited, and more animals need to be used for future studies.

**Table 1.** Means, standard error of BW, and morphometrics of male (*n =* 12 heads).

Age	BW (kg)	WH (cm)	BL (cm)	CG (cm)	HH (cm)	Chest width (cm)
0	64.58 ± 9.00	74.42 ± 3.03	66.50 ± 4.17	78.75 ± 4.22	75.83	22.00 ± 2.56
30	70.50 ± 9.50	82.99 ± 5.49	78.02 ± 10.84	87.42 ± 4.58	88.23 ± 9.27	25.58 ± 1.38
60	100.23 ± 8.47	88.32 ± 4.87	83.15 ± 9.91	97.17 ± 4.73	94.22 ± 7.68	27.67 ± 1.72
90	128.76 ± 6.30	92.65 ± 4.95	87.85 ± 8.70	104.83 ± 6.85	100.61 ± 8.84	29.58 ± 1.31
120	154.08 ± 9.95	97.94 ± 4.73	93.74 ± 9.80	110.83 ± 7.15	103.06 ± 4.58	31.75 ± 1.22
150	177.43 ± 12.34	102.95 ± 5.48	98.82 ± 10.03	118.33 ± 5.96	108.39 ± 4.63	33.08 ± 1.16
180	199.74 ± 19.03	107.30 ± 5.01	104.56 ± 7.83	124.42 ± 43.8	112.67	34.17 ± 1.75
210	218.15 ± 15.52	108.95 ± 4.92	109.52 ± 7.67	130.50 ± 3.61	114.83 ± 3.72	35.08 ± 2.27
240	237.83 ± 25.99	109.88 ± 3.68	114.95 ± 6.16	135.50 ± 5.13	116.82 ± 4.12	36.25 ± 2.09
270	261.54 ± 30.95	112.89 ± 3.64	116.91 ± 5.91	140.42 ± 6.35	119.04 ± 3.46	37.42 ± 2.35
300	283.14 ± 37.15	113.72 ± 3.24	118.71 ± 6.95	143.75 ± 7.28	120.45 ± 4.77	37.67 ± 2.74

**Table 2.** Means, standard error of BW, and morphometrics of female (*n =* 14 heads).

Age	BW (kg)	WH (cm)	BL (cm)	CG (cm)	HH (cm)	Chest width (cm)
0	66.36 ± 9.30	72.07 ± 3.97	64.21 ± 4.23	78.21 ± 6.00	73.50 ± 7.05	20.36 ± 4.29
30	68.57 ± 6.24	80.37 ± 6.16	70.99 ± 8.66	84.5 ± 6.51	85.24 ± 5.64	24.57 ± 5.88
60	96.86 ± 7.83	87.88 ± 5.14	85.03 ± 5.78	96.29 ± 5.50	93.84 ± 5.69	28.71 ± 5.04
90	123.79 ± 7.44	93.25 ± 5.83	89.57 ± 5.63	104.07 ± 5.37	98.65 ± 6.04	31.50 ± 4.00
120	146.95 ± 11.19	98.20 ± 6.14	94.98 ± 7.35	113.21 ± 4.98	102.64 ± 4.06	32.57 ± 3.61
150	172. 41 ± 13.85	103.37 ± 5.51	100.04 ± 8.38	118.64 ± 5.12	108.02 ± 4.60	34.14 ± 4.45
180	168.11 ± 77.47	107.49 ± 4.74	104.80 ± 6.97	124.79 ± 5.58	112.41 ± 4.39	34.79 ± 4.42
210	206.48 ± 24.40	109.10 ± 4.89	108.65 ± 6.62	130 ± 4.76	113.92 ± 4.67	35.79 ± 4.71
240	221.20 ± 24.30	111.28 ± 4.22	111.92 ± 5.81	133.93 ± 5.99	116.71 ± 4.03	37.14 ± 5.02
270	238.56 ± 27.64	113.46 ± 5.14	115.39 ± 6.89	137.07 ± 6.63	117.59 ± 4.68	37.57 ± 5.11
300	252.41 ± 29.86	114.77 ± 5.29	117.73 ± 8.37	141.14 ± 8.05	120.12 ± 4.32	38.86 ± 4.94

**Table 3.** Stepwise regression of BW and morphometrics in BB X FH.

Variables in model	Model	*R* ^2^	AIC
BL + CG	BW = −190.24 + 0.87 BL + 2.35 CG	0.83	1943.25
WH + BL + CG	BW = −200.07 + 0.36 WH + 0.75 BL + 2.22 CG	0.83	1944.41
BL + CG + CW	BW = −191.30 + 0.88 BL + 2.25 CG + 0.35 CW	0.83	1944.84
WH + BL + CG + HH	BW = −197.28 + 0.49 WH + 0.82 BL + 2.24 CG – 0.23 HH	0.83	1946.14
WH +BL + CG + CW	BW = −200.15 + 0.33 WH + 0.77 BL + 2.15 CG + 0.28 CW	0.83	1946.14
CG	BW = −185.27 + 3.04 CG	0.82	1952.16
BL	BW = −168.21 + 3.44 BL	0.76	2035.88
WH	BW = −278.19 + 4.47 WH	0.75	2045.90
HH	BW = −265.74 + 4.13 HH	0.73	2067.25
CW	BW = −120.34 + 890 CW	0.61	2174.68

**Table 4.** Random forest summary for predicting BW using morphometrics data.

mtry	*R* ^2^	RMSE	MAE
2	0.86	26.35	16.76
3	0.85	27.03	17.09
5	0.83	28.45	17.76

## Conclusion

In conclusion, random forest produces a better model than stepwise regression. The random forest analysis produced the best predictive performance, indicating that this modeling approach is optimal for calculating BW in this investigation. Stepwise regression analysis revealed that the models involving BL and CG are the most effective for estimating the BW of crossbred cattle. The CG is a simple equation that can be used to estimate BW.
